# Kisspeptin and neurokinin B interactions in modulating gonadotropin secretion in women with polycystic ovary syndrome

**DOI:** 10.1093/humrep/deaa104

**Published:** 2020-06-08

**Authors:** Karolina Skorupskaite, Jyothis T George, Johannes D Veldhuis, Robert P Millar, Richard A Anderson

**Affiliations:** d1 MRC Centre for Reproductive Health, The Queen’s Medical Research Institute, University of Edinburgh, 47 Little France Crescent, Edinburgh EH16 4TJ, UK; d2 Warwick Medical School, Coventry CV4 7AL, UK; d3 Boehringer Ingelheim, Bracknell RG12 8YS, UK; d4 Endocrine Research Unit, Center for Translational Science Activities, Mayo Clinic, Rochester, MN 55905, USA; d5 Centre for Neuroendocrinology and Mammal Research Institute, University of Pretoria, 0028 Pretoria, South Africa; d6 Institute for Infectious Diseases and Molecular Medicine, University of Cape Town, 7925 Observatory, South Africa

**Keywords:** neurokinin B / kisspeptin / LH pulsatility / PCOS / GnRH

## Abstract

**STUDY QUESTION:**

What is the role of the hypothalamic neuropeptide neurokinin B (NKB) and its interaction with kisspeptin on GnRH/LH secretion in women with polycystic ovary syndrome (PCOS)?

**SUMMARY ANSWER:**

Administration of neurokinin 3 receptor antagonist (NK3Ra) for 7 days reduced LH and FSH secretion and LH pulse frequency in women with PCOS, whilst the stimulatory LH response to kisspeptin-10 was maintained.

**WHAT IS KNOWN ALREADY:**

PCOS is characterized by abnormal GnRH/LH secretion. NKB and kisspeptin are master regulators of GnRH/LH secretion, but their role in PCOS is unclear.

**STUDY DESIGN, SIZE, DURATION:**

The NK3Ra MLE4901, 40 mg orally twice a day, was administered to women with PCOS for 7 days (*n* = 8) (vs no treatment, *n* = 7). On the last day of NK3Ra administration or the equivalent day in those not treated, women were randomized to 7-h kisspeptin-10 (4 µg/kg/h i.v.) or vehicle infusion. This was repeated with the alternate infusion in a subsequent cycle.

**PARTICIPANTS/MATERIALS, SETTING, METHODS:**

Subjects were women with PCOS, studied in a Clinical Research Facility. Reproductive hormones were measured before and after NK3Ra administration. On the last day of NK3Ra administration (or the equivalent cycle day in untreated women), all women attended for an 8-h frequent blood sampling to allow analysis of the pulsatile LH secretion.

**MAIN RESULTS AND THE ROLE OF CHANCE:**

NK3Ra reduced LH secretion (4.0 ± 0.4 vs 6.5 ± 0.8 IU/l, *P* < 0.05) and pulse frequency (0.5 ± 0.1 vs 0.8 ± 0.1 pulses/h, *P* < 0.05); FSH secretion was also reduced (2.0 ± 0.3 vs 2.5 ± 0.4 IU/l, *P* < 0.05). Without NK3Ra pre-treatment, kisspeptin-10 increased LH secretion (5.2 ± 0.5 to 7.8 ± 1.0 IU/L, *P* < 0.05), with a positive relationship to oestradiol concentrations (*r*^2^ = 0.59, *P* < 0.05). After NK3Ra administration, the LH response to kisspeptin-10 was preserved (vehicle 3.5 ± 0.3 vs 9.0 ± 2.2 IU/l with kisspeptin-10, *P* < 0.05), but the positive correlation with oestradiol concentrations was abolished (*r*^2^ = 0.07, ns. after NK3Ra). FSH secretion was increased by kisspeptin-10 after NK3Ra treatment, but not without NK3Ra treatment.

**LIMITATIONS, REASONS FOR CAUTION:**

The study did not explore the dose relationship of the effect of NK3R antagonism. The impact of obesity or other aspects of the variability of the PCOS phenotype was not studied due to the small number of subjects.

**WIDER IMPLICATIONS OF THE FINDINGS:**

These data demonstrate the interactive regulation of GnRH/LH secretion by NKB and kisspeptin in PCOS, and that the NKB system mediates aspects of oestrogenic feedback.

**STUDY FUNDING/COMPETING INTEREST(S):**

Wellcome Trust through Scottish Translational Medicine and Therapeutics Initiative (102419/Z/13/A) and MRC grants (G0701682 to R.P.M. and R.A.A.) and MR/N022556/1 to the MRC Centre for Reproductive Health. This work was performed within the Edinburgh Clinical Research Facility. J.T.G. has undertaken consultancy work for AstraZeneca and Takeda Pharmaceuticals and is an employee of Boehringer Ingelheim. R.P.M. has consulted for Ogeda and was CEO of Peptocrine. R.A.A. has undertaken consultancy work for Merck, Ferring, NeRRe Therapeutics and Sojournix Inc. J.D.V. and K.S. have nothing to disclose.

**TRIAL REGISTRATION NUMBER:**

N/A.

## Introduction

Polycystic ovary syndrome (PCOS) is the most common endocrinopathy in women of reproductive age and the leading cause of anovulatory infertility (Sirmans and Pate, 2013). The clinical syndrome is characterized by the presence of some or all of (i) chronic anovulation with oligo/amenorrhoea; (ii) clinical and/or biochemical hyperandrogenaemia; and/or (iii) polycystic appearance of the ovaries (Rotterdam, 2004; Teede *et al.*, 2018). It is a heterogenous condition with multifactorial pathophysiology, including genetic, environmental, neuroendocrine and metabolic components, although those underpinnings and their relative importance remain unclear (Goodarzi *et al.*, 2011; Hayes *et al.*, 2015). Treatment is essentially symptomatic, and further insight into the mechanisms underlying the central endocrinopathy has potential for the development of new therapies.

Women with PCOS often show a neuroendocrine disturbance, with increased LH pulse secretion, with little effect on FSH secretion, presumably reflecting a similar perturbation in GnRH neuronal activity (Santen and Bardin, 1973; Rebar *et al.*, 1976; Marshall *et al.*, 2001; Moore and Campbell, 2017). This LH hyper-pulsatility contributes to increased thecal androgen secretion and failure of ovulation, constituting a pivotal pathogenic role in the syndrome (Marshall *et al.*, 2001; Caldwell *et al.*, 2017). Kisspeptin–neurokinin B (NKB) pathways have emerged as master regulators of GnRH and LH secretion (Pinilla *et al.*, 2012; Skorupskaite *et al.*, 2014; Clarkson *et al.*, 2017). Patients with loss-of-function mutations in kisspeptin, NKB and their respective receptors (KISS1R and NK3R) show pubertal delay (de Roux *et al.*, 2003; Seminara *et al.*, 2003; Topaloglu *et al.*, 2009; Topaloglu *et al.*, 2012), whereas precocious puberty is seen in those with activating mutations in KISS1R (Teles *et al.*, 2008). In a randomized controlled trial, neurokinin 3 receptor antagonist (NK3Ra) administration in women with PCOS decreased the frequency of LH pulses (indicative of pulsatile GnRH release) and also LH and testosterone concentrations (George *et al.*, 2016). This is in concordance with a suppressive action of NKB antagonism on pulsatile LH secretion shown in postmenopausal women with hot flushes (Skorupskaite *et al.*, 2018b), in a model of the mid-cycle LH surge in healthy women (Skorupskaite *et al.*, 2016) and in gonadectomized animals (Fraser *et al.*, 2015; Li *et al.*, 2015). These findings clearly illustrate NKB modulation of LH secretion via GnRH, and it is possible that dysregulation of NKB signalling may play a role in the neuroendocrine pathogenesis of PCOS.

NKB signalling is closely interlinked with hypothalamic kisspeptin pathways, with overlapping expression in hypothalamic neurones impinging on GnRH neurones (Clarkson and Herbison, 2006; Goodman *et al.*, 2007; Hrabovszky *et al.*, 2010). Where these neurones also express the opioid dynorphin, they are termed KNDy neurones. The functional interactions of these pathways are incompletely determined. In patients with inactivating mutations in NKB (*TAC*) and its receptor (*TAC3*), with slow GnRH secretion, exogenous kisspeptin-10 restored LH pulse frequency (Young *et al.*, 2013). Kisspeptin-10 stimulated LH secretion after it was decreased during NK3Ra administration in healthy men (Skorupskaite *et al.*, 2017). This and concordant data from animal models have led to the conclusion that NKB signalling is functionally upstream of kisspeptin in regulating GnRH secretion (Billings *et al.*, 2010; Corander *et al.*, 2010; Garcia-Galiano *et al.*, 2012; Navarro *et al.*, 2011). However, in postmenopausal women with high LH output, the gonadotropin response to kisspeptin-10 before and after pharmacological blockage of NK3R was limited (Skorupskaite *et al.*, 2018b). Whilst these data support an overall hierarchy whereby NKB is functionally afferent to kisspeptin, the interactions between those hypothalamic neuropeptides in modulating GnRH/LH secretion are unclear. There are no data on this interaction in women with PCOS, a human disease model of LH hyper-secretion.

Kisspeptin neurones are critical in mediating the effects of sex-steroid feedback on GnRH secretion (Dhillo *et al.*, 2007; Jayasena *et al.*, 2011; Chan *et al.*, 2012; George *et al.*, 2012). The LH response to kisspeptin is greatest in the late follicular phase of the menstrual cycle (Dhillo *et al.*, 2007; Jayasena *et al.*, 2011; Chan *et al.*, 2012) and is positively related to circulating oestradiol levels (Narayanaswamy *et al.*, 2016a; Skorupskaite *et al.*, 2016). However, this relationship between kisspeptin-induced LH response and oestradiol concentrations is disrupted with NKB antagonism in healthy women, indicating a key role of NKB in modulating the effect of the sex-steroid environment (Skorupskaite *et al.*, 2016). Despite clear roles for NKB and kisspeptin in regulating LH secretion, the relative contribution of the NKB-kisspeptin pathway to the pathophysiology of PCOS remains uncertain, and may have therapeutic potential. We therefore hypothesized that NK3R antagonism would affect LH secretion in women with PCOS through modification of aspects of its pulsatile secretion, and that a functionally upstream site of action would not prevent the stimulatory effect of kisspeptin on LH secretion, but that the LH response to kisspeptin would be modified, indicating a role of NK3R signalling in the regulation of that response to circulating oestradiol levels.

## Materials and Methods

### Participants

Ten otherwise healthy women with PCOS, aged 19–31 years, with a body mass index of 20–40 kg/m^2^ and a last menstrual period 2–7 months ago, were recruited into the study (Table I); all provided informed written consent. Five of the women participated in both arms (NK3Ra treatment and no treatment group) of the study, thus there were a total of 15 cycles in the study (8 with NK3Ra and 7 without treatment). Subjects needed to meet at least two of the following Rotterdam criteria after exclusion of related disorders of menstrual irregularity: (i) oligomenorrhoea/amenorrhoea for at least 1 year; (ii) clinical and/or biochemical signs of hyperandrogenism (above laboratory normal of total testosterone i.e. >1.9 nmol/l, measured by LC-MS) or Free Androgen Index >6.5); and/or (iii) polycystic ovaries (≥12 follicles per ovary and/or ovarian volume ≥10 ml) documented on high resolution transvaginal ultrasound scan (Rotterdam, 2004). All subjects in the study were oligo/amenorrhoeic (see Table I for phenotypes). Participants were not taking any hormonal contraception nor had an intrauterine device *in situ*. They otherwise had a normal physical examination, and their full blood count, renal function and electrolytes, liver function and electrocardiogram were within normal limits.

**Table I:** Baseline characteristics of the women with PCOS enrolled in the study.

Subject	Age (years)	BMI (kg/m^2^)	Months since LMP	LH (IU/l)	FSH (IU/l)	Oestradiol (pmol/l)	Testosterone (nmol/l)	FAI	PCOS phenotype
1	31	38	7	17.9	6.6	241	1.9	7.9	B
2	30	30	2	11.6	5.8	95	0.9	7.5	A
3	30	44	14	16.4	6.9	218	1.8	18	A
4	24	40	8	6.7	4.9	180	1.9	9.5	A
5	19	35	3	15.9	6.9	250	2.4	7.3	B
6	30	20	2	12	3	273	1.8	1.9	A
7	21	38	2	9.7	3.8	265	1.1	4.2	D
8	26	33	5	7.8	3.9	152	2.4	6.9	B
9	28	36	3	6.1	3.2	126	1.3	8.7	A
10	27	34	12	5.7	3.9	167	1	6.3	B
**Mean ± SEM**	27 ± 1.3	35 ± 2.1	6 ± 1.4	11.0 ± 0.4	4.9 ± 0.5	197 ± 19.5	1.7 ± 0.2	7.8 ± 1.3	

All women were oligo/amenorrhoeic. Phenotype A = oligo/amenorrhoea, clinical or biochemical hyperandrogenism and polycystic ovaries; Phenotype B = oligo/amenorrhoea and hyperandrogenism; Phenotype D = oligo/amenorrhoea and polycystic ovaries. Reference range for testosterone, 0.3–1.9 nmol/l, and for FAI (free androgen index), <6.5.

FAI, free androgen index; LMP, last menstrual period; NK3Ra, neurokinin 3 receptor antagonist; PCOS, polycystic ovary syndrome.

### Study drugs

Medroxyprogesterone acetate (Pfizer, Surrey, UK) was used to induce withdrawal menstruation. Kisspeptin-10 was custom synthesized under GMP standards (Bachem GmBH, Weil am Rhein, Germany) (George *et al.*, 2011), and 1 mg kisspeptin-10 was dissolved in 5 ml sterile normal (0.9%) saline immediately before infusion. The syringe and line for infusion were first coated for 30 min with kisspeptin-10 to minimize peptide loss from adherence to the plastic. Sterile normal saline was infused as vehicle. The NK3Ra MLE4901 (previously termed AZD4901, Astra-Zeneca, Macclesfield, UK) was administered orally at 40 mg twice daily. This dose of MLE4901 reduced LH secretion in normal women and in women with PCOS (George *et al.*, 2016; Skorupskaite *et al.*, 2016).

### Protocol

**Figure 1:**
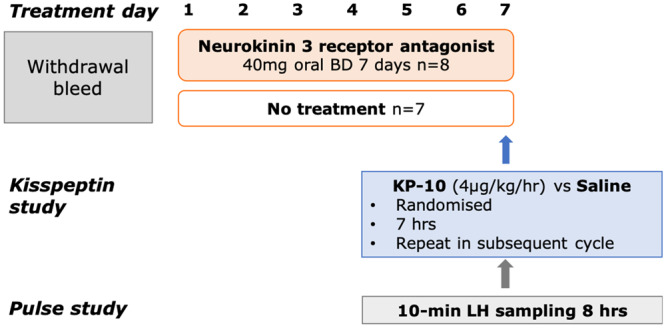
**Study protocol diagram.** Following medroxyprogesterone withdrawal menstruation, in eight treatment cycles, women received neurokinin 3 receptor antagonist (NK3Ra; MLE4901) 40 mg oral twice daily starting from Cycle day 1–2 for 7 days, and in 7 cycles, women received no treatment. On Day 6 or 7 of NK3Ra dosing in the treatment group and the equivalent day in the no treatment group, women were randomized to 7 h kisspeptin-10 or vehicle infusion, returning for alternate infusion after subsequent medroxyprogesterone induced menstruation. Reproductive hormones were measured throughout the study and LH pulsatility was assessed during 10-min frequent blood sampling for 8 h on the infusion day.

Medroxyprogesterone acetate, 10 mg twice daily orally for 7 days, was administered to induce a withdrawal bleed. In eight treatment cycles, women received NK3Ra (MLE4901) 40 mg oral twice daily starting from Cycle day 1–2 for 7 days, and in seven cycles, they received no treatment (Fig. 1). Volunteers attended the clinical research facility on Day 6 or 7 of NK3Ra dosing (Cycle day 6–8) in the treatment group and on the equivalent day in the no treatment group. After an hour of baseline sampling, volunteers were randomized (using sealed envelopes) to receive a continuous intravenous infusion of kisspeptin-10 (4 µg/kg/h) or vehicle for 7 h. In the NK3Ra treatment group, the last dose of MLE4901 was taken on the morning of kisspeptin-10 or vehicle administration. All women returned to receive the alternate infusion of kisspeptin-10 or vehicle after at least a month wash out period and repeated induction of menstruation with medroxyprogesterone followed by administration of NK3Ra or no treatment, as they have received in the previous cycle.

### Blood sampling and hormone assays

Peripheral venous blood was sampled between 8 and 9 am for LH, FSH and oestradiol in the treatment group on the day of commencing NK3Ra. Seven days later on the last day of NK3Ra administration (or equivalent cycle day in the untreated women), all women attended for an 8-h visit (starting 8–9 am), blood samples were collected via an indwelling i.v. cannula at 10 min intervals for assessments of LH pulsatile secretion; FSH and oestradiol were measured hourly.

Blood samples were centrifuged at 4°C for 10 min at 3000 rpm and serum was frozen at −20°C or below until analysis. LH and FSH were measured by ELISA as previously described (George *et al.*, 2011) 17β-oestradiol was measured on a Roche Cobas E411 immunoassay automated analyser (Roche Diagnostics, Burgess Hill, UK).

The number of LH pulses, secretory mass of LH per pulse, and the basal (non-pulsatile) and pulsatile (integral of dual amplitude and frequency regulation) LH secretion were identified by an established deconvolutional algorithm with cluster analysis (93% sensitivity and specificity) blinded to treatment allocation, and ApEn was quantified as a measure of secretory regularity (Veldhuis *et al.*, 2008; Liu *et al.*, 2009).

### Statistical analysis

The study size was determined from similar investigations of LH pulsatile secretion (George *et al.*, 2011, 2012). Mean LH, FSH and oestradiol concentrations pre- and post-NK3Ra treatment were compared using the Student’s paired t-test.

Mean hourly hormone concentrations during 8 h of kisspeptin-10 or vehicle infusion were compared using two-way ANOVA with repeated measures followed by Bonferroni’s *post hoc* multiple comparisons test. Pearson’s correlation coefficient was computed to assess the relationship between oestradiol concentrations and LH response to kisspeptin-10 with and without the NK3Ra.

Characteristics of LH pulsatile secretion were compared using Student’s t-test or Wilcoxon-matched pairs-signed rank test for paired data and unpaired t-test or Mann–Whitney test for unpaired data, depending on the normality of distribution of the data.

Data are presented as mean ± SEM. Differences were regarded as significant at a two-sided *P* < 0.05. The statistical software package GraphPad Prism (GraphPad, San Diego, CA, USA) was used.

### Ethical approval

The study protocol was approved by South East Scotland Research Ethics Committee (Ref: 09/S1101/67) and all women gave consent, in writing.

## Results

### NK3Ra decreases gonadotropin secretion

**Figure 2:**
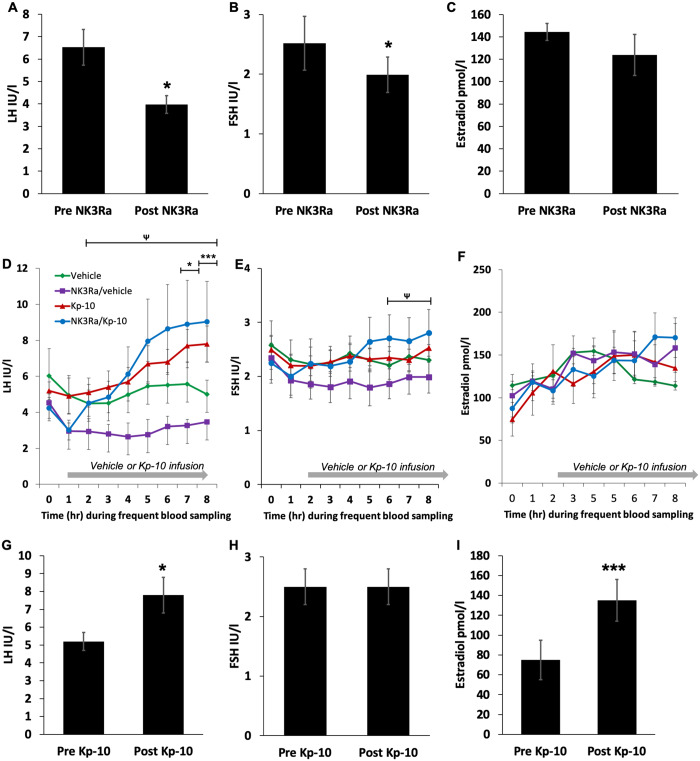
**Reproductive hormone response to administration of NK3Ra or no treatment followed by kisspeptin/vehicle infusion cycles in women with polycystic ovary syndrome.** (**A–C**) Mean LH (**A**), FSH (**B**) and oestradiol (**C**) concentrations at the end of NK3Ra treatment comparted to pre-treatment day (*n* = 8). (**D–F**) Time course analysis of frequent blood sampling of LH (**D**), FSH (**E**) and oestradiol (**F**) levels before (Time 0) and during 7 h of vehicle or kisspeptin-10 infusion in the treatment (NK3Ra) group (*n* = 8) and no treatment group (*n* = 7). Kisspeptin-10 stimulated LH secretion in both vehicle and NK3Ra-treated women (*P* < 0.05) but did not affect FSH secretion. Serum oestradiol levels were higher after kisspeptin-10 administration compared to pre-treatment concentrations (*P* < 0.001), although were not different compared to that after infusion with vehicle. LH levels were lower in NK3Ra compared with vehicle-treated women (*P* < 0.0001), although Bonferroni’s *post hoc* multiple comparison test found no significant changes at specific time points. (**G** and **H**) Mean LH (**G**), FSH (**H**) and oestradiol (**I**) concentrations at the end of kisspeptin-10 treatment comparted to pre-treatment (*n* = 7). Data are presented as mean ± SEM. *P < 0.05; ***P < 0.001. Mean differences at specific time points for vehicle versus kisspeptin-10 infused in no NK3Ra-treated women: *P < 0.05, ***P < 0.001. For vehicle vs kisspeptin-10 in NK3Ra-treated women: ^Ψ^*P* < 0.05.

NK3Ra decreased LH concentrations from 6.5 ± 0.8 IU/l pre-treatment to 4.0 ± 0.4 IU/l after 7 days of NK3Ra administration (*P* < 0.05) (Fig. 2A). Analysis of LH at hourly intervals for 8 h after the last NK3Ra dose also showed that overall LH secretion was lower in NK3Ra-treated women compared to no treatment (*P* < 0.0001, Fig. 2D), although *post hoc* analysis indicated no significant differences in LH levels at any individual hourly time point.

Serum FSH levels were reduced with NK3Ra administration when compared to pre-treatment concentrations (pre-NK3Ra 2.5 ± 0.4 vs post-NK3Ra 2.0 ± 0.3 IU/l, *P* < 0.05) (Fig. 2B). Analysis of FSH at hourly intervals for 8 h after the last dose showed that overall FSH secretion was also lower with NK3Ra treatment compared to no treatment (*P* < 0.0001, Fig. 2E). Oestradiol concentrations were unaffected by the NK3Ra (Fig. 2C and F).

### Effects of kisspeptin-10 on gonadotropin secretion

Kisspeptin-10 stimulated LH secretion, which was sustained throughout 7 h of kisspeptin-10 administration (*P* < 0.05) (Fig. 2D). Kisspeptin-10 increased LH secretion from 5.2 ± 0.5 IU/l pre-infusion to 7.8 ± 1.0 IU/l at the end of infusion (*P* < 0.05) (Fig. 2G), compared to 5.0 ± 0.8 IU/l after infusion with vehicle (*P* < 0.001). FSH secretion was unaffected by kisspeptin-10 (Fig. 2E and H).

Serum oestradiol levels were higher after kisspeptin-10 administration compared to pre-treatment concentrations (pre-infusion 75 ± 20 vs post-infusion 135 ± 21 pmol/l, *P* < 0.001) (Fig. 2I), although they were not different compared to concentrations after infusion with vehicle (7 h of kisspeptin-10: 135 ± 21 vs vehicle 114 ± 27 pmol/l, ns.) (Fig. 2F).

### Effect of NK3Ra on kisspeptin-10 induced gonadotropin secretion

Following treatment with NK3Ra, kisspeptin-10 also stimulated LH release (end of kisspeptin-10: 9.0 ± 2.2 vs vehicle 3.5 ± 0.3 IU/l, *P* < 0.05, Fig. 2D). This increase was similar to LH concentrations stimulated with kisspeptin-10 infusion alone (end of kisspeptin-10 with NK3Ra: 9.0 ± 2.2 vs kisspeptin-10 alone 7.8 ± 1.0 IU/l, ns., Fig. 2D).

In the presence of the NK3Ra, kisspeptin-10 stimulated FSH secretion (end of kisspeptin-10 infusion 2.8 ± 0.4 IU/l vs 2.2 ± 0.4 pre-infusion and 2.0 ± 0.3 with vehicle, both *P* < 0.05, Fig. 2E).

### NK3Ra abolishes oestradiol dependency of kisspeptin-10 response

**Figure 3:**
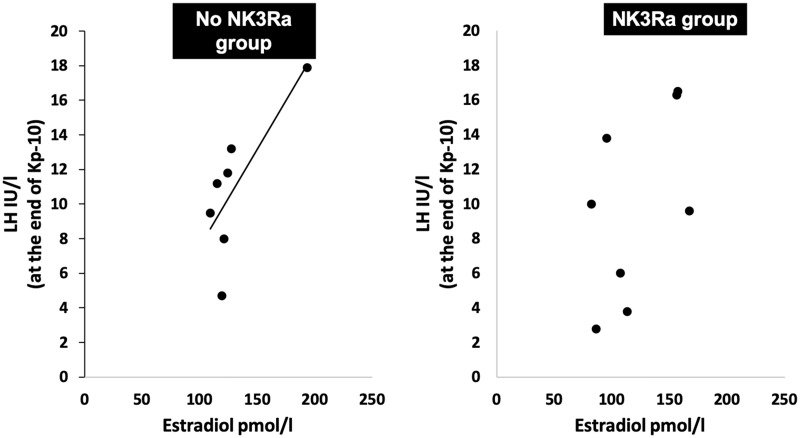
**Correlation between oestradiol concentrations and LH response to kisspeptin-10 in with and without NK3Ra.** Kisspeptin-10 response on LH secretion was positively related to oestradiol levels (*r*^2^ = 0.59, *P* < 0.05), whilst this was not seen with NK3Ra treatment (*r*^2^ = 0.07, ns).

The effect of NK3Ra in the relationship between the LH response to kisspeptin-10 and oestradiol exposure was investigated by analysing LH concentration at the end of kisspeptin-10 infusion in relation to oestradiol concentration at the start of the kisspeptin-10 infusion. There was a strong positive correlation between LH response to kisspeptin-10 and oestradiol concentration in women not treated with the NK3Ra (*r*^2^ = 0.59, *P* < 0.05) (Fig. 3). However, in NK3Ra-treated women, the LH response to kisspeptin-10 showed no such relationship (*r*^2^ = 0.07, ns.).

### Interaction between kisspeptin-10 and NK3Ra in regulation of LH pulsatile secretion

**Figure 4:**
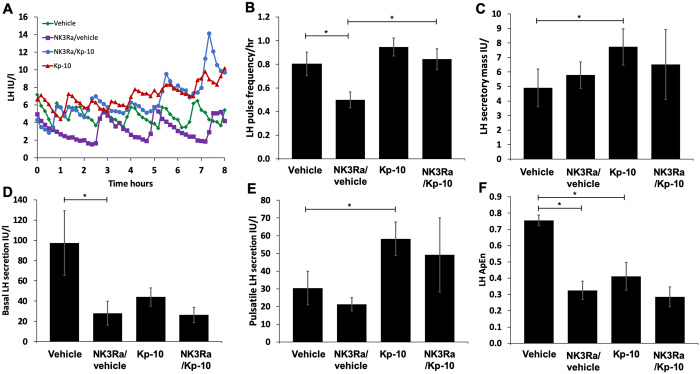
**Analysis of 8-h secretory patterns during vehicle and kisspeptin-10 infusion with and without NK3Ra.** (**A**) Illustrative LH pulse profile from one subject undergoing vehicle (green diamonds), NK3Ra (purple squares), kisspeptin-10 (red triangles) and NK3Ra followed by kisspeptin-10 (blue circles) treatment visits. Mean LH pulse frequency (**B**), secretory mass of LH per pulse (**C**), basal (non-pulsatile) LH secretion (**D**), pulsatile LH secretion (**E**) and the relative orderliness/regularity of LH secretory pattern (**F**) during vehicle and kisspeptin-10 infusion with (*n* = 8) and without (*n* = 7) pre-treatment with NK3Ra. The NK3Ra significantly reduced LH pulse frequency. While Kp-10 alone did not affect LH pulse frequency, it did increase it in the presence of NK3Ra. Pulsatile LH secretion and LH secretory mass were increased by Kp-10, but not affected by NK3Ra. Basal LH secretion was reduced by NK3Ra, but was not stimulated by Kp-10 alone or with NK3Ra. Both NK3Ra and Kp-10 increased the relative orderliness/regularity of LH secretory pattern (i.e. reduced ApEn). Mean ± SEM. *P < 0.05.

Exemplars of LH pulse profiles during vehicle and kisspeptin-10 infusion with and without NK3Ra treatment are shown in Fig. 4A. LH pulse frequency was decreased to 0.5 ± 0.1 pulses/h after NK3Ra treatment compared to 0.8 ± 0.1 pulses/h in the no treatment group (*P* < 0.05) (Fig. 4A and B). Kisspeptin-10 alone had no effect on LH pulse frequency, but in women treated with NK3Ra, administration of kisspeptin-10 increased LH pulse frequency to 0.8 ± 0.1 pulses/h (*P* < 0.05 vs NK3Ra with vehicle infusion) (Fig. 4B).

NK3Ra antagonist alone did not reduce secretory mass of LH per pulse (Fig. 4C). Secretory mass per pulse was increased during infusion of kisspeptin-10 compared with vehicle (*P* < 0.05) but not following pre-treatment with the NK3Ra (Fig. 4C).

Basal LH secretion was also decreased with NK3Ra treatment (*P* < 0.05 vs vehicle in the no treatment group, Fig. 4D) but there was no effect on pulsatile LH secretion (Fig. 4E). Kisspeptin-10 increased pulsatile but not basal LH secretion (*P* < 0.05 vs vehicle, Fig. 4D and E). Kisspeptin-10 infusion in NK3Ra-treated women induced no change in basal or pulsatile LH secretion.

The regularity of LH secretory pattern was assessed by approximate entopy (ApEn). Both NK3Ra and kisspeptin-10 infusion imposed greater orderliness (lower ApEn) in LH secretion (*P* < 0.05, Fig. 4F) with no additional change with the combination of treatments.

### Tolerability and safety

The NK3Ra, MLE4901, was well tolerated with no treatment discontinuations. Haematology and biochemistry safety parameters remained stable in all subjects throughout the study period.

## Discussion

We, here, investigated the interaction between kisspeptin and NKB in the modulation of pulsatile GnRH/LH secretion in women with PCOS, to explore the neuroendocrine component of the pathophysiology of this syndrome. Pharmacological blockage of NKB-NK3R signalling decreased both LH and FSH secretion and slowed LH pulsatility. Although kisspeptin-10 stimulated LH release to a similar degree with and without the NK3Ra treatment, the strong relationship between oestradiol and LH response to kisspeptin was abolished in the presence of the NK3Ra.

These data confirm that NK3Ra treatment in women with PCOS reduced LH secretion, indicative of suppressed hypothalamic GnRH pulsatile secretion. This observation is consistent throughout animal studies (Fraser *et al.*, 2015; Li *et al.*, 2015) and in healthy women in states of fast and slow LH pulse frequency and in varying sex-steroid environments (George *et al.*, 2016; Skorupskaite *et al.*, 2016, 2018a,b). In a randomized double blinded, placebo-controlled clinical trial in women with PCOS administering the same NK3Ra at the same dose and for the same 7 day duration, LH pulse frequency was reduced, as were LH secretion and testosterone secretion, whilst FSH levels remained unchanged (George *et al.*, 2016). Here, we show suppressed FSH secretion on Day 7 of NK3Ra treatment assessed over 8 h of frequent blood sampling post dose, which may have been missed when single time-point FSH concentrations were compared at baseline and post treatment in previous studies. This is consistent with FSH secretion being modulated by non-pulsatile GnRH release as basal LH secretion was also suppressed by the NK3Ra. In women with PCOS, NK3R antagonism had no detected effect on oestradiol concentrations, which is in contrast to healthy women administered this drug during the follicular phase of the menstrual cycle, where reduced oestradiol secretion was thought to have increased (or prevented a decrease) in FSH release (Skorupskaite *et al.*, 2018a). The absence of a detected change in oestradiol concentrations may reflect the dysregulated follicular development characteristic of PCOS as well as the limited changes in LH and FSH, and the short duration of treatment.

Kisspeptin-10 stimulated LH secretion in women with PCOS, although this effect was modest compared to the response to the same dose in healthy women under high oestrogen exposure (Skorupskaite *et al.*, 2016). Although obesity can be associated with blunted LH pulse amplitude, and all the participants in the study but one were obese, this study does not adequately explore this association. Likewise, kisspeptin-54 was ineffective in stimulating LH secretion in normal women in the early follicular and luteal phases, despite eliciting a marked response in the preovulatory phase (Dhillo *et al.*, 2007; Chan *et al.*, 2012; Jayasena *et al.*, 2011). A close relationship between the oestrogenic environment and the response to kisspeptin was shown here with a direct positive relationship with oestradiol concentrations, thus this relationship is present in women with PCOS as well as in normal women (Narayanaswamy *et al.*, 2016a; Skorupskaite *et al.*, 2016). However, the relationship between oestradiol concentrations and the LH response to kisspeptin-10 was absent during NK3Ra treatment, as we have previously reported in normal women (Skorupskaite *et al.*, 2016). The LH hypersecretion which is characteristic of PCOS is thought to be driven by oestrogen levels (Rebar *et al.*, 1976). These data therefore indicate that a key aspect of the NKB component of KNDy neurone function in both normal women and those with PCOS is to sense and modulate the effect of the ambient oestrogenic signal, and thus determine the kisspeptin signal to the GnRH neurone. This is likely to be of importance in determining the precise regulation of LH secretion, which is so critical for the regulation of ovarian steroidogenesis and follicle development.

The stimulatory effect of kisspeptin-10 on LH secretion was preserved during NK3Ra treatment, despite partially suppressed LH concentrations. Intriguingly, these data also showed stimulation of FSH secretion by kisspeptin-10 during NK3Ra treatment, but not by kisspeptin infusion without NK3R antagonism. An FSH response to kisspeptin has been minimal and inconsistent in previous studies (Dhillo *et al.*, 2007; Jayasena *et al.*, 2011; Chan *et al.*, 2012; Skorupskaite *et al.*, 2014). The background reduced GnRH secretion pattern might have allowed a stimulatory effect to be detected with the detailed blood sampling protocol used here. That NKB signalling is functionally upstream of kisspeptin has been previously demonstrated in patients with inactivating mutations in the NKB-NK3R pathway as well as in animal studies (Billings *et al.*, 2010; Corander *et al.*, 2010; Navarro *et al.*, 2011; Ramaswamy *et al.*, 2011; Garcia-Galiano *et al.*, 2012; Young *et al.*, 2013). This relationship is maintained in PCOS, with high endogenous GnRH/LH pulsatile secretion compared to the reduced pulsatile secretion in patients with NKB/NK3R inactivating mutations. However, having performed an array of clinical studies in healthy men and women in different states of LH pulsatile secretion and sex-steroid environment using a consistent protocol of NK3R antagonism for 7 days and exogenous kisspeptin-10 administration (Skorupskaite *et al.*, 2016, 2017, 2018a,b) (Tables II and III), we demonstrate a more complex interaction between those hypothalamic neuropeptides than a simply direct interaction. In men, LH secretion was decreased with the NK3Ra, but this did not affect an immediate stimulatory LH response to kisspeptin (Skorupskaite *et al.*, 2017). In contrast, the hypothalamo-pituitary-gonadal (HPG) axis in hypo-oestrogenic postmenopausal women was refractory to any manipulation by kisspeptin and NK3Ra, suggesting that the loss of negative oestrogen feedback has an overriding impact (Skorupskaite *et al.*, 2018b). That NK3Ra abolished the positive correlation between serum oestradiol and LH response to kisspeptin in women PCOS suggests an additional level of interaction between kisspeptin and NKB; similarly, in healthy women, under high exogenous oestrogen exposure, NK3Ra shortened the LH response to kisspeptin-10 and disrupted the relationship between the kisspeptin effect and oestradiol concentrations (Skorupskaite *et al.*, 2016). Indeed, the orderliness and regularity of LH secretion was here shown to be increased with administration of either kisspeptin-10 or NK3Ra in women with PCOS. A potential inhibitory action of NKB itself was suggested in healthy men, as the stimulatory LH response to co-infusion of kisspeptin-54 and NKB was significantly lower than with kisspeptin alone (Narayanaswamy *et al.*, 2016b); however we have found no evidence of a stimulatory effect of NK3R antagonism in any of the human models thus far studied.

**Table II:** Summary table showing effects of KP-10 and NK3Ra (MLE4901) administration, by a consistent protocol, on gonadotropin secretion in different states of LH pulsatility and sex-steroid environments in men and women.

	Men	Postmenopausal women	Premenopausal women	Women with PCOS
Study	Skorupskaite *et al.* (2017)	Skorupskaite *et al.* (2018b)	Skorupskaite *et al.* (2016)	Present data
LH status	Normal	Very high	LH surge model	High
Sex-steroid feedback	Negative	Loss of negative	Negative then positive	Dysregulated
NK3Ra response	Decreased LH	Marginal LH decrease	Marginal decrease in basal LH	Decreased LH
	Decreased FSH	No FSH effect	Increased FSH	Decreased FSH
KP-10 response	Increased LH	No LH/FSH effect	Increased LH and FSH	Increased LH
				No FSH effect
KP-10 and NK3Ra (vs KP-10 alone)	No change	No LH/FSH effect	Shorter KP response	Increased LH and FSH
			Loss of relationship with oestradiol	Loss of relationship with oestradiol

**Table III:** Effects on NK3R antagonism, kisspeptin-10 infusion and their combination on various parameters of pulsatile nature of LH, and by inference GnRH secretion, in healthy men and women at different stages of reproductive life.

Group	Men	Postmenopausal women—with hot flushes	Premenopausal women—early follicular phase	Premenopausal women—LH surge model			Women with PCOS		
Study	Skorupskaite *et al.* (2017)	Skorupskaite * et al.* (2018b)	Skorupskaite *et al.* (2018a)	Skorupskaite *et al.* (2016)			Present data		
Treatment/Pulsatility parameter	NK3Ra	NK3Ra	NK3Ra	NK3Ra	KP-10	NK3Ra and KP-10	NK3Ra	KP-10	NK3Ra and KP-10
LH pulse frequency	No change	Decrease	No change	Decrease	Increase	Increase	Decrease	No change	Increase
LH mass per pulse	No change	Increase	No change	No change	Increase	Increase	No change	Increase	No change
Basal LH	Decrease	Decrease	Decrease	No change	Decrease	No change	Decrease	No change	No change
Pulsatile LH	Decrease	No change	No change	No change	Increase	Increase	No change	Increase	No change
ApEn (irregularity)	Decrease	No change	No change	Decrease	Decrease	Decrease	Decrease	Decrease	No change

In addition to the overall fall in LH secretion during treatment with NK3Ra, we demonstrate a reduction in the frequency of LH pulses as well as in basal LH secretion, implying that similar changes in the pattern of GnRH release were also achieved. These findings are consistent with low LH pulsatile secretion observed in patients with genetic defects leading to impaired NKB signalling (Young *et al.*, 2013) and in a randomized controlled trial in women with PCOS administering the same NK3Ra (George *et al.*, 2016). Antagonism of NKB signalling appears to suppress LH pulse frequency uniformly in states associated with increased LH pulsatility, such as in women during the late follicular phase (Skorupskaite *et al.*, 2016) and in postmenopause (Skorupskaite *et al.*, 2018b) and in gonadectomized animals (Fraser *et al.*, 2015; Li *et al.*, 2015), whereas no effect is seen in states of slower LH pulse frequency, such as in women during the early follicular phase (Skorupskaite *et al.*, 2018a) and in men (Skorupskaite *et al.*, 2017) (Table III). This suggests that these states of higher LH pulse frequency may be driven by NKB signalling, with this pathway having less importance at lower frequencies. NK3Ra also improved the orderliness and regularity of LH pulses in women with PCOS, which was also seen with kisspeptin-10 for the first time. Dysregulation and hypersecretion of LH associated with PCOS may derive from disordered hypothalamic kisspeptin and/or NKB secretion and their downstream effects on the pattern of GnRH release, suggesting new paradigms in the neuropathophysiology of PCOS.

Kisspeptin-10 increased the mass of LH released per pulse and the overall mass of LH secreted in a pulsatile pattern in women with PCOS, consistent with increased LH concentrations. Overall, the effects of kisspeptin-10 on pulsatile GnRH/LH secretion in women with PCOS were similar to those seen in healthy women during exogenous oestradiol-induced LH surge, apart from the increased LH pulse frequency in the latter (Skorupskaite *et al.*, 2016) (Table III). However, kisspeptin-10 increased LH pulse frequency after the pulses were slowed down by pre-treatment with the NK3Ra. Similarly, to the above considerations regarding a higher LH pulse frequency being needed for NKB antagonism to suppress LH pulsatility, there may be a threshold of LH pulse frequency beyond which further stimulatory effects of kisspeptin cannot be achieved, in the presence of steroidal feedback. Similarly, infusion of kisspeptin-10 restored low LH pulse frequency in men and women with inactivating mutations in NKB signalling (Young *et al.*, 2013). Although this is consistent with the overall hierarchy whereby NKB is functionally upstream of kisspeptin, co-administration of NK3Ra prevented kisspeptin-induced changes in the pattern of LH and inferred GnRH release (Table III), further indicating the complex interactions between those hypothalamic neuropeptides.

Reduced LH secretion through NK3R antagonism seen in this study may be of therapeutic application in women with PCOS. In a short-duration randomized controlled trial of this approach, serum testosterone levels were reduced (George *et al.*, 2016) but trials of sufficient duration to see clinical benefit have not yet been reported. The reduction in LH hypersecretion with NK3R antagonism may be of value for the induction of ovulation in women with PCOS, although the small reduction in FSH reported here may also impact on that. The phenotypic diversity of women with this condition, particularly in relation to lean/obese status, is also very relevant and was not explored in this study, due to the small number of women included. Kisspeptin administration has been explored in women with PCOS to induce ovulation (Romero-Ruiz *et al.*, 2019) and to reduce the risk of ovarian hyperstimulation syndrome in assisted reproduction (Abbara *et al.*, 2015).

The work described above forms part of series of reproductive neuroendocrine studies in humans (Skorupskaite *et al.*, 2016, 2017, 2018a,b). The small number of subjects is an important limitation, although they have been studied using consistent protocols and randomisation. The complexity of the study has precluded the involvement of large patient numbers to adequately explore the possibility that some phenotypes of PCOS may show different responses. Five women with PCOS out of the ten recruited participated in all four arms of the studies which may further limit the generalisability of the data. Although a specific PCOS phenotype was not selected for this study, our data show a degree of consistency of response that does not indicate relevance to only certain subgroups.

The KNDy system is a key mediator of the effects of sex steroids and other, e.g. metabolic, factors on GnRH secretion and thus the reproductive system. The present data suggest a specific role for NKB in mediating the influence of circulating oestrogen levels, but in the context of PCOS, it will be important to investigate the influence of obesity on kisspeptin and NKB modulation of GnRH secretion Anti-Mullerian hormone (AMH) has also been implicated as a regulator of GnRH secretion (Cimino *et al.*, 2016), and there may be effects of long-term increased hypothalamic exposure to AMH and oestradiol on the kisspeptin/NKB system and its interaction with other hypothalamic neurotransmitter pathways (Moore and Campbell, 2016).

In summary, kisspeptin-10 infusion in women with PCOS increased LH secretion, with a direct relationship to oestradiol exposure. NK3R antagonism reduced LH secretion and pulsatility, and whilst the LH response to kisspeptin-10 was preserved, its relationship with circulating oestradiol concentrations was not. Although kisspeptin-10 increased the frequency of LH pulses, changes in other parameters of LH secretory pattern were prevented when co-administered with the NK3Ra. These data thus indicate that NKB and kisspeptin regulate GnRH secretion in women with PCOS and that there is a complex rather than purely direct interaction between those neuropeptides in determining the precise pattern and degree of GnRH/LH secretion. Whilst these data show largely similar effects to those seen in normal women, the differences in both the effects of NK3R antagonism and response to kisspeptin-10 may be attributable to the precise level of sex-steroid feedback, and do not indicate major differences in the neuroendocrine regulation of LH secretion in PCOS at the level of the KNDy neurone.
